# Case report: Hereditary sensory autonomic neuropathy presenting as bifid deformity to the tongue

**DOI:** 10.3389/fdmed.2023.1179795

**Published:** 2023-10-19

**Authors:** Kelsey O’Hagan-Wong, Dana Smith, Hernan Gonorazky, Michael Casas

**Affiliations:** ^1^ Department of Dentistry, University of Toronto, Toronto, Canada; ^2^ Department of Dentistry, The Hospital for Sick Children, University of Toronto, Toronto, ON, Canada

**Keywords:** pediatric dentistry, congenital insensitivity to pain, hereditary sensory autonomic neuropathy, tongue ulceration, bifid deformity of the tongue

## Abstract

Hereditary sensory autonomic neuropathy (HSAN) is a group of rare genetic disorders in which affected patients have a diminished capacity to feel pain. Patients with HSAN may present with a wide range of factitial injuries, where injury to the oral cavity may be an early presenting sign. While existing literature on HSAN is scant, many reports highlight the long-term outcomes that may include enucleation of eyes, amputation of fingers and limbs, and disfigurement of the tongue. This case describes a five-month-old female with repetitive injury to the tongue causing it to heal with a bifid deformity. The patient was later diagnosed with HSAN type 4. This case highlights the importance of recognition of extensive oral trauma as one of the early signs of HSAN that should provoke a timely referral for neurological assessment.

## Introduction

Hereditary sensory autonomic neuropathy (HSAN) is a group of rare genetic disorders that are characterized by the inability to perceive noxious stimuli (1). HSAN is divided into eight subtypes based on clinical phenotype (2). The pathophysiology of HSAN has been linked to more than 12 genes (3, 4). HSAN Type 4 is characterized by the inability to feel pain or thermal stimuli as well as anhidrosis and intellectual disability. The pattern of inheritance for HSAN Type 4 is autosomal recessive with approximately 50 per cent of cases associated with parental consanguinity (5). Its prevalence is estimated to be 1/600,000–950,000 (5). Patients with HSAN are susceptible to a variety of physical injuries that can lead to permanent disability. The clinical manifestations of HSAN generally appear in infancy and include self-mutilating injuries to the oral cavity, eyes, and hands (6, 7). Repetitive bone fractures can also lead to Charcot joints and osteomyelitis. Intellectual disability, anhidrosis, and lack of olfaction are also common phenotypes (symptoms) of HSAN. Affected children present with a higher incidence of *staphylococcus aureus* infections and delayed wound healing (8). As a result of complications related to injuries and fractures, patients with more severe presentations of HSAN rarely survive into adulthood.

There is no cure for HSAN and management is focused on the prevention of physical injuries and the subsequent complications to those injuries. Diagnosis of HSAN early in life coupled with increased attentiveness to injury prevention and medical surveillance may improve long-term health and quality of life. The literature highlights the potentially devastating effects of HSAN when affected patients are not closely followed by a medical team. In this report, we present the case of a female infant who initially presented with repeated self-mutilation of the tongue and was diagnosed with HSAN Type 4 at 10 months of age.

## Case description

A 5-month female patient presented to the Emergency Department at The Hospital for Sick Children with a chief complaint of bleeding from the tongue. The patient was the first child of healthy, non-consanguineous parents and had no other comorbid conditions. The patient was born full term and her birth height and weight were within normal limits. The parents reported that the child's first four months of life were uneventful until her primary mandibular incisor teeth erupted.

**Figure 1:**
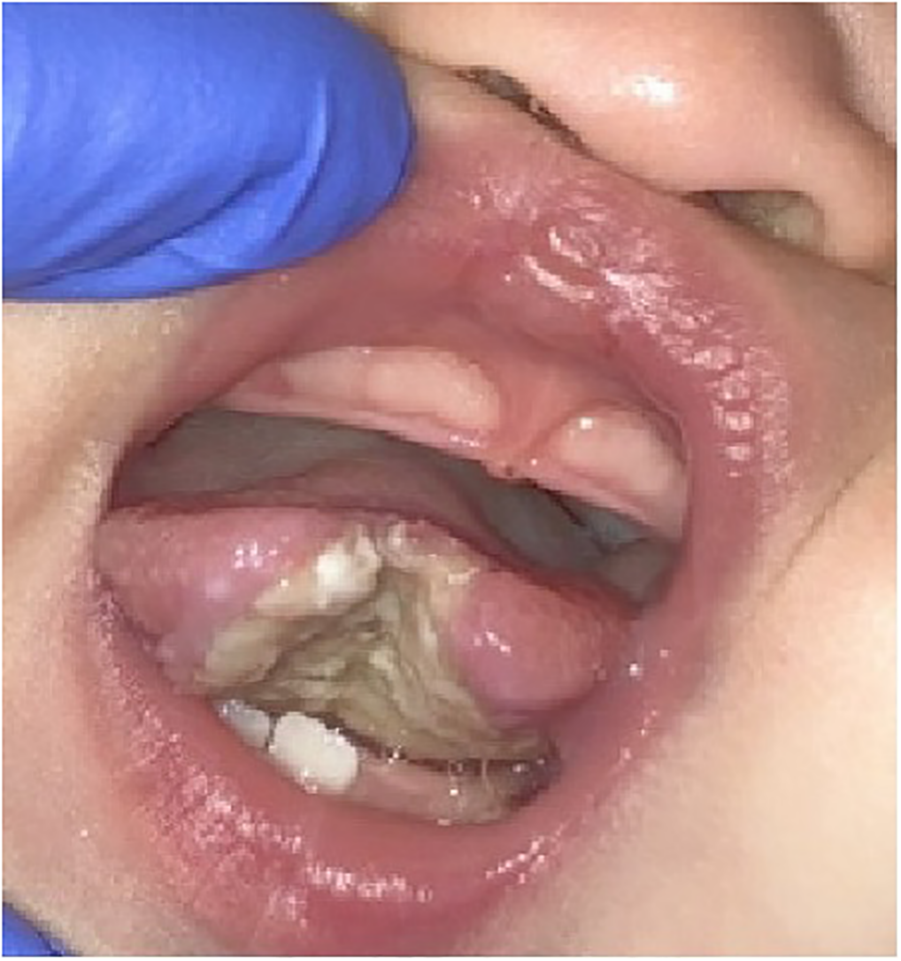
Intraoral photo showing ulceration of the tongue following the eruption of the mandibular primary central incisors.

Clinical examination revealed partially erupted mandibular primary central incisors as well as a large ulceration on the ventral surface with significant tissue loss at the midline (Figure 1). The dorsal surface of the tongue was within normal limits. The child was eating and feeding normally. A provisional diagnosis of traumatic ulcerative granuloma with stromal eosinophilia was made at the time of the emergency department assessment.

The parents were provided with recommendations for non-invasive management of the tongue ulcerations such as using a pacifier to prevent the tongue from interfering with the teeth and changes to how the infant was fed to avoid trauma to the tongue. Reduction of the incisal edge of the incisors or application restorations have been offered as temporary solution with a low likelihood of success. Intraoral appliances such as bite guards and splints have been used in patients with pathological chewing secondary to brain trauma (9). In this case, secure retention of the appliance would be compromised by the very small number of teeth to aid with mechanical retention of a bite guard or splint. Extraction of the mandibular incisors was discussed but the parents opted for modifications to nursing bottles, pacifier use and follow-up in the Dental Clinic in one-week.

**Figure 2:**
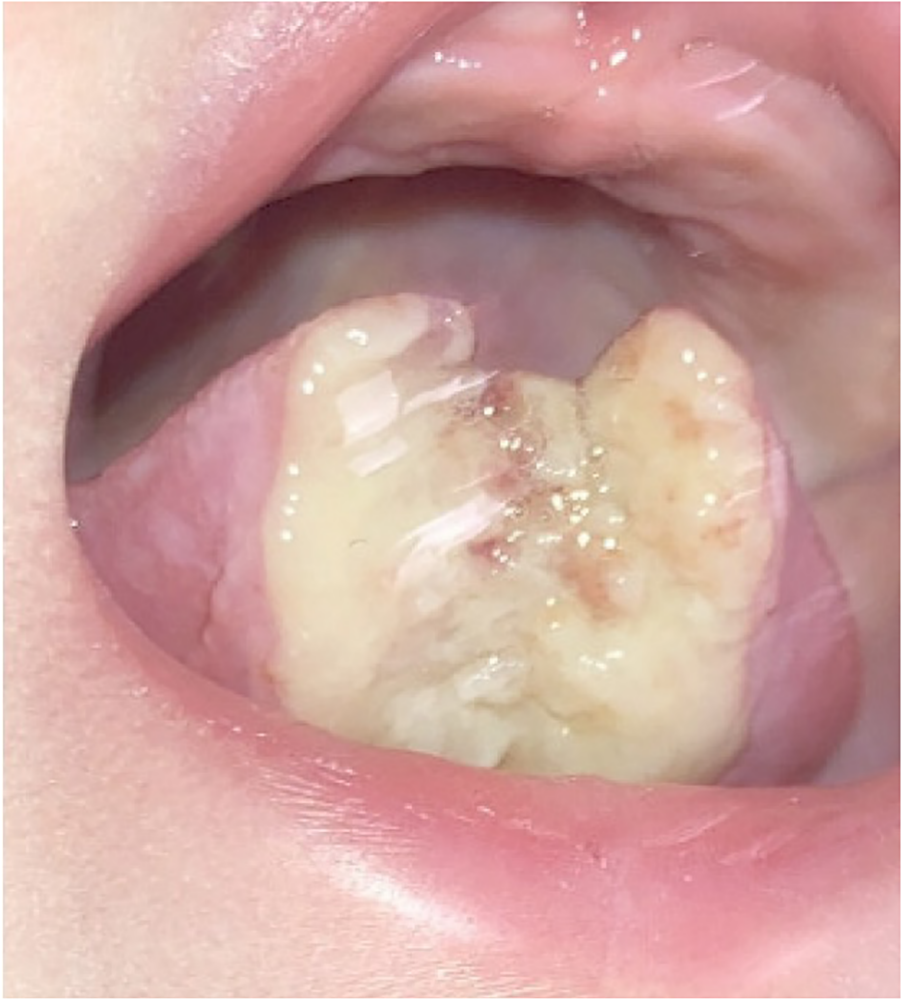
Intraoral photo of the tongue showing persistent ulceration of the tongue at the one-week follow-up visit.

No apparent signs of healing were noted at the one-week follow-up (Figure 2). The parents reported that the patient did not appear to be in pain and was still feeding and sleeping normally. A diagnosis of Riga Fede disease was made. Riga Fede disease (syndrome) is “an ulceration of the ventral surface of the tongue, often caused by repetitive traumatic injuries due to backward and forward movements of the tongue over the mandibular anterior incisors” (10). Extraction of the mandibular primary central incisors was recommended. The parents declined extraction in anticipation of laboratory results from their child's pediatrician who had undertaken blood work to assess for underlying medical conditions.

**Figure 3:**
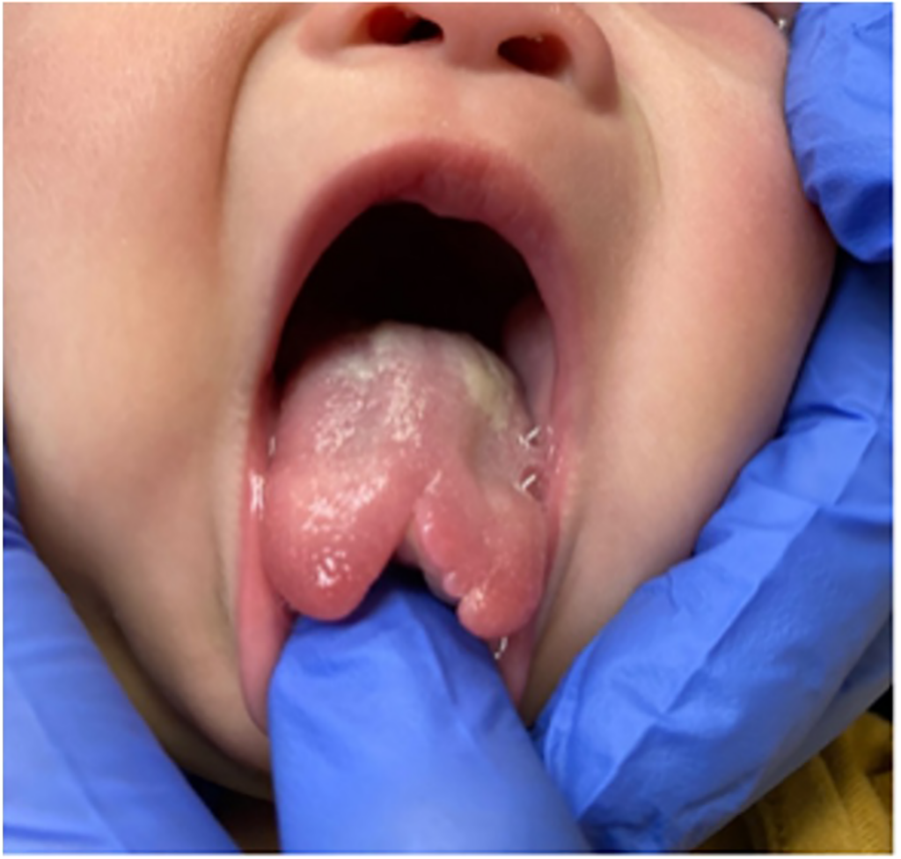
Intraoral photo showing the permanent bifid deformity of tongue healing four weeks after the extraction of mandibular primary incisors.

Four weeks after the initial emergency room visit, after the blood work was determined to be normal, the parents consented to the extraction of the mandibular primary central incisors. A week postoperatively, the extraction site healing was uneventful, and the tongue ulceration demonstrated signs of resolution. A midline defect involving the tip of the tongue was noted (Figure 3). The parents were cautioned that further trauma to the tongue was likely to occur with the eruption of additional teeth.

**Figure 4:**
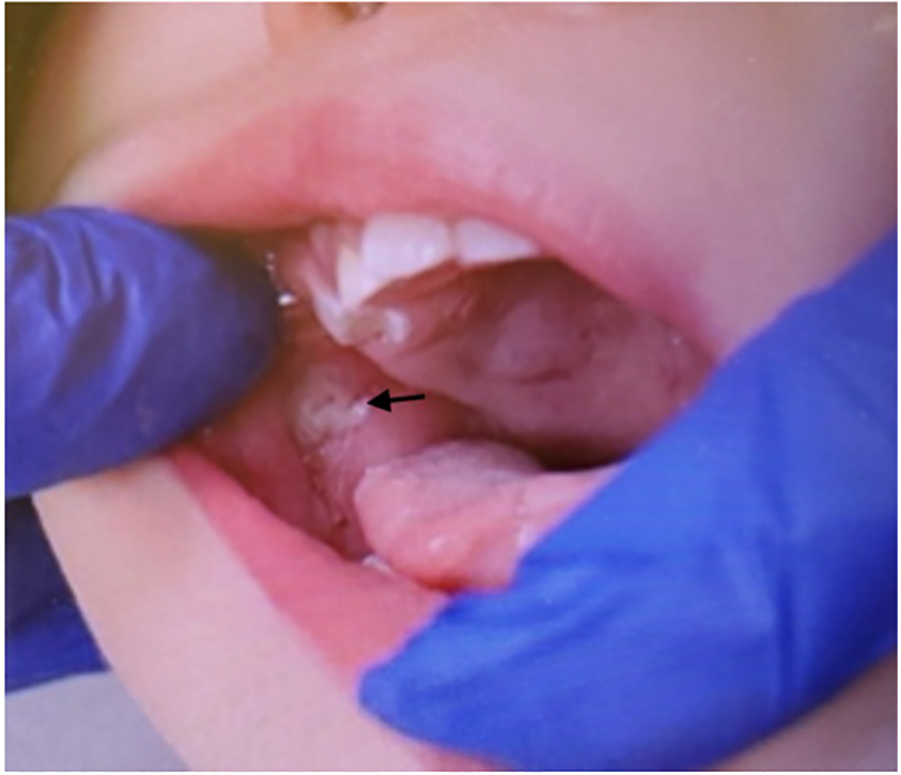
Intraoral photo of recurrent ulcerations on the tongue and buccal mucosa following eruption of posterior primary molars.

Three months later, the patient presented to the Dental Clinic with partially erupted maxillary central and mandibular lateral primary incisors. A new traumatic tongue ulcer measuring 0.5 cm on the right dorsal surface and tip of the tongue as well as buccal ulcerations were present (Figure 4). The parents declined extractions at this time. Given the extent and recurrent nature of the oral injuries, the patient was referred for neurological testing.

The Division of Neurology administered sensory testing and a nerve conduction study. The resultant report indicated that the patient had a decreased response to temperature and to electrical shock. The sympathetic skin response was also abnormal. A provisional diagnosis of hereditary sensory and autonomic neuropathy was made and genetic testing recommended. A molecular HSAN genetic testing demonstrated that the patient had two pathological variants in the *NTRK1* gene (c.2066C>T (p.Pro689Leu), c.851-33T>A (intronic) compound heterozygous), resulting in faulty or deficient neurotrophic tyrosine receptor type 1. This protein is found on certain cells, especially nerve cells that transmit pain, temperature, and touch sensations. These genetic results are consistent with a diagnosis of HSAN type 4. Due to the autosomal recessive nature of the disease, genetic testing was also performed on the parents to confirm carrier status. The parents were informed of the other clinical manifestations of the disease including intellectual disability, anhidrosis, and decreased lacrimation. The patient was 10 months of age when the diagnosis of HSAN Type 4 was made.

Shortly after the diagnosis of HSAN Type 4, the parents consented to extractions of the mandibular primary lateral incisors. The parents expressed concern over the esthetic appearance of the bifid tongue and whether it would interfere with their child's speech. The patient was referred to the Division of Plastic Surgery for consultation with regard to surgical correction of the tongue deformity. Due to the risk of continued traumatic injury to the tongue with eruption of additional primary teeth, it was recommended that correction be deferred until the child is 5–6 years of age.

The parents indicated that no other injuries, bruises, or cuts were noted anywhere else on the patient's body. The patient's developmental milestones were all normal and no signs of intellectual deficits or developmental delay were noted. Assessment in the Department of Ophthalmology detected no traumatic eye injuries. Currently, the patient's parents have declined further extractions. Follow-up appointments continue.

## Discussion

HSAN Type 4 is a rare genetic disorder which is characterized by the impairment of pain sensation leading to self-mutilating behaviors. This case of HSAN Type 4 was diagnosed at 10 months of age after repeated episodes of self-mutilation to the tongue. Following the eruption of primary incisors, the patient repeatedly traumatized the tongue causing it to heal with a bifid deformity, which to our knowledge has not yet been previously reported. It is important to note that during periods of extensive tongue ulceration, the patient did not appear to show any signs of discomfort or difficulty feeding. This lack of reaction to such severe oral injuries should prompt the clinician to pursue further clinical investigations specifically neurological assessment.

Initially, this patient's oral condition was managed primarily as Riga Fede disease. Dental interventions to prevent Riga Fede disease referenced in the literature include smoothing sharp edges of teeth, placement of composite resin or other restorative materials to sharp edges of teeth, intraoral appliances (such as mouth guards), and extraction of primary teeth (9).

It is plausible that earlier extraction of the mandibular primary central incisors may have mitigated the damage to the tongue. Although recommended on multiple occasions, the family declined extractions. Studies that examined parental decision-making preferences of ill children suggest that parental perception of urgency can sometimes differ from that of the medical team. Specifically, when a child is given a significant medical diagnosis, the parent is more likely to view treatment and management as urgent (11). Therefore, it is plausible to infer that the parents would have agreed to definitive treatment options if the diagnosis of HSAN had been made earlier.

At present, the tongue cleft defect is being monitored by the Division of Plastic Surgery and will be surgically repaired when the patient is at an age when she is less likely to re-traumatize the tongue. The surgical correction of median tongue clefts can yield satisfactory result in cases of congenital tongue clefts (12). The outcome for remediation of a traumatically induced tongue cleft deformity is not certain.

The strength of this current case report lies in the relatively early diagnosis of this rare syndrome. The diagnosis of HSAN at ten months of age prompted dental interventions paired with routine follow up visits with dentistry, ophthalmology, and occupational therapy for counselling and injury surveillance, with the hope of preventing long term complications of the disease. Limitations of this report include no long-term follow up of this case to build on the success of her dental and oral growth and general development. There is no consensus on best dental management practices for patients with HSAN. The limited pool of patients with HSAN creates an obstacle for clinicians to offer a consensus on the most suitable treatment options.

## Conclusion

To our knowledge this case is the first describing a bifid tongue as a presenting feature of HSAN4. Oral trauma is one of the earliest symptoms of the disease and pediatric dentists may be one of the first healthcare providers to assess the condition. Early diagnosis of HSAN may potentially allow for interventions that mitigate more extensive injuries.

## Data Availability

The raw data supporting the conclusions of this article will be made available by the authors, without undue reservation.
